# The optimal mechanical condition in stem cell-to-tenocyte differentiation determined with the homogeneous strain distributions and the cellular orientation control

**DOI:** 10.1242/bio.039164

**Published:** 2019-05-22

**Authors:** Yasuyuki Morita, Toshihiro Sato, Kouji Higashiura, Yusho Hirano, Fuga Matsubara, Kanau Oshima, Koji Niwa, Yuhki Toku, Guanbin Song, Qing Luo, Yang Ju

**Affiliations:** 1 Department of Micro-nano Mechanical Science and Engineering, Graduate School of Engineering, Nagoya University, Furo-cho, Chikusa-ku, Nagoya 464-8603, Japan; 2 Key Laboratory of Biorheological Science and Technology, Ministry of Education, College of Bioengineering, Chongqing University, Chongqing 400044, China

**Keywords:** Differentiation, Human bone marrow-derived mesenchymal stem cell (hBMSC), Mechanical stimulus, Tendon, Tenocyte, Tissue engineering

## Abstract

In tendon tissue engineering, mechanical stimulus-induced differentiation is one of the most attractive techniques for stem cell-to-tenocyte differentiation in terms of cost, safety and simplicity. However, the most effective strain amplitude for differentiation using cyclic stretching remains unknown. Existing studies have not constrained cell reorientation behavior during cyclic stretching, resulting in uncertainty regarding the loads experienced by cells. In addition, strain distribution homogeneity of the culture membrane is important. Here, we improved the strain distribution uniformity of the membrane and employed a microgrooved membrane to suppress cell reorientation. Then we evaluated the most effective strain amplitude (0, 2, 4, 5, 6, or 8%) for the differentiation of mesenchymal stem cells into tenocytes by measuring mRNA expression levels. The maximum expression of all tenogenic markers was observed at a 5% strain. These results contribute to tendon tissue engineering by clarifying the most effective strain amplitude during tenogenic differentiation induction using cyclic stretching.

## Abstract (precis)

**Summary:** We determined the relationship between the cyclic strain of culture membrane and cellular gene expression, from which the most effective strain for tenogenic differentiation of stem cells was elucidated.

## INTRODUCTION

Tendon is a critical tissue responsible for bone-muscle joint stability and body locomotion, but it has minimal self-curing abilities due to a lack of cells and feeding vessels. Spontaneous cure often results in the formation of scar tissue, which differs morphologically, biochemically and biomechanically from healthy tendon tissue (Sharma and Maffulli, 2006). Repaired tendon tissue exhibits impaired dynamic properties and strength (Tohyama et al., 2003); thus, current treatments for tendon injury, both conservative and surgical, are limited in their effectiveness (Bagnaninchi et al., 2007).

The lack of effective treatments for tendon injury suggests a need for tendon tissue engineering, in which functional tissue produced *in vitro* by stem cells that have proliferative and multilineage potentials is implanted in the body. Pittenger et al. (1999) showed that mesenchymal stem cells (MSCs) from human bone marrow with high proliferative abilities could differentiate into tenocytes. This finding initiated research on tendon tissue engineering with tenogenic differentiation of MSCs. Recently, several techniques have been reported to promote differentiation, including biochemical excitation (Hankemeier et al., 2005), mechanical stimulus (Riehl et al., 2012), co-culturing (Schneider et al., 2011) and culturing on scaffolds (Sahoo et al., 2006). Details of tenogenic differentiation methods and clinical applications can be found in reviews by Liu et al. (2017) and Lui et al. (2011).

As a mechanical stimulus, cyclic uniaxial stretching is a promising tool for the induction of differentiation in tendon tissue engineering in terms of safety, cost and simplicity, and several studies have aimed to determine the most effective strain amplitude for tenogenic differentiation of MSCs (Liu et al., 2017; Lui et al., 2011). For example, using cyclic stretching as a stimulus, Chen et al. (2008) determined 10% and Nam et al. (2015) determined 8% to be the most effective strain conditions for the tenogenic differentiation of MSCs. Similarly, our group determined approximately 8% to be the most effective strain amplitude (Morita et al., 2013b, 2014). However, these results do not necessarily represent optimal strain conditions, since these studies only examined two or three amplitudes, which were selected arbitrarily. Therefore, our group developed a new elastic chamber for cell culturing with a non-uniform deformation field, which exerts a continuous strain gradient on the cell-culturing membrane (Morita et al., 2013c), to more accurately determine the optimal strain condition for tenogenic differentiation of MSCs (Morita et al., 2015).

As another limitation, none of the aforementioned studies constrained cellular reorientation, which reduces strain. For example, when cyclic stretching is applied to MSCs cultured on an elastic poly(dimethylsiloxane) (PDMS) membrane, the MSCs realign to a specific angle (approximately ±60–90° to the stretch direction) to reduce longitudinal strain, where each MSC is considered to be an ellipsoid (Morita et al., 2013b, 2014; Wang et al., 1995), which is a type of cellular avoidance reaction (Buck, 1980). Therefore, because they permitted reorientation behavior, previous studies could not accurately determine the optimal strain amplitude for stem cell-to-tenocyte differentiation, since MSCs could face any preferable direction to avoid cyclic stretch loading. Thus, techniques that force cells to orient parallel to the stretch direction and suppress the stretch avoidance reaction are required to elucidate the most effective strain amplitude for tenogenic differentiation of MSCs. Several research groups have applied scaffold materials, such as aligned poly(lactide-co-glycolide) nanofiber scaffolds (Subramony et al., 2013) and decellularized tendon scaffolds (Youngstrom et al., 2015), to improve cellular orientation for tenogenic differentiation. Moreover, a simple elastic membrane (e.g. PDMS) would be useful to clarify the differentiation characteristics with respect to the cyclic stretching load, which could enable the exclusion of additional factors. As a breakthrough, Wang et al. (2004) invented a PDMS membrane with aligned microgrooves, and succeeded in constraining cellular reorientation. Furthermore, they determined the most effective strain amplitude and the cyclic stretch orientation of the microgrooved membrane to upregulate tendon protein production of human tendon fibroblasts (Li et al., 2004; Wang et al., 2005; Yang et al., 2004). Microgrooved membranes, which suppress cellular reorientation, could be useful for clarifying the effective strain conditions for tenogenic differentiation of MSCs.

In the present work, we analyzed the effect of strain amplitude on tenogenic differentiation of human bone marrow-derived MSCs (hBMSCs) with cyclic stretching using an aligned microgrooved membrane to inhibit cellular reorientation and apply a homogeneous strain load to the aligned cells. To determine the most effective strain condition, the uniformity of the strain distribution over the cell-culturing area on the aligned microgrooved membrane must be ensured. Therefore, we developed an elastic PDMS chamber with aligned microgrooves to reduce the non-uniformity of the applied strain distribution. Six possible strain amplitudes [0 (control), 2, 4, 5, 6 and 8% strain] were applied to promote differentiation. Expressed mRNAs of tenogenic differentiation markers were assessed under each strain condition using quantitative real-time reverse-transcription (qRT)-PCR to determine the most effective strain condition. This study is unique in that the results accurately show the most effective strain condition for stem cell-to-tenocyte differentiation induction in tendon tissue engineering. Moreover, the procedure presented herein represents a powerful tool to optimize mechanical conditions in tissue engineering.

## RESULTS AND DISCUSSION

### Improved strain distribution homogeneity of the membrane of the newly developed chamber

**Fig. 1.**
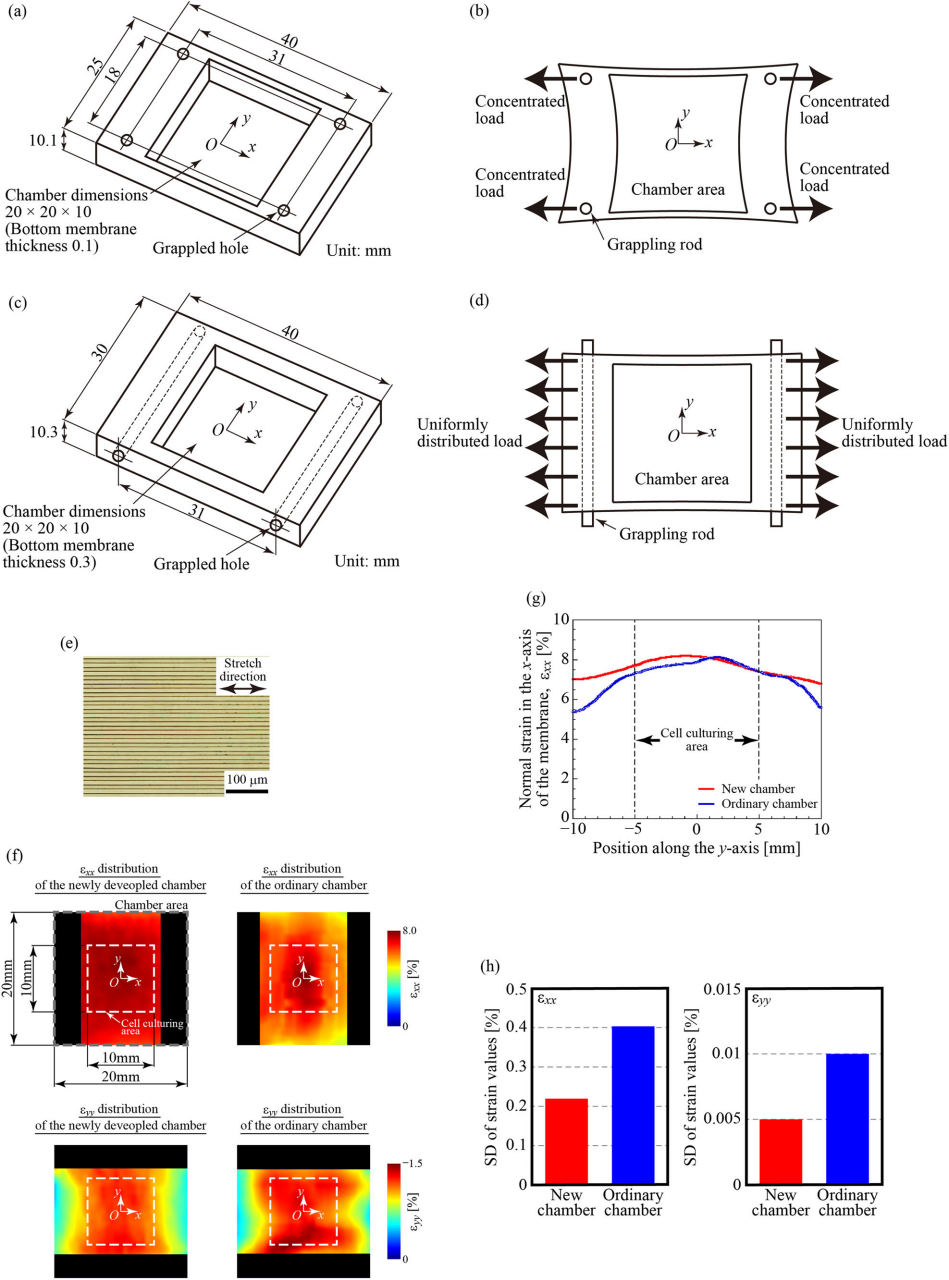
**Newly developed elastic chamber for cell culturing with cyclic stretching.** (A) A commercially available PDMS chamber with thin sidewalls (2.5 mm, one side), in which the load can be applied from four points of support. (B) The commercial chamber takes the form of a bobbin due to the concentrated load through the four grappling rods and contraction due to Poisson's effect. (C) The newly developed PDMS chamber has thicker sidewalls (5 mm, one side) and can apply a uniformly distributed load, which (D) can reduce contraction caused by Poisson's effect and the bobbin-like deformation. The deformations in B and D are exaggerated for illustrative purposes. (E) The fabricated PDMS membrane has aligned microgrooves, which are 10 µm wide and 5 µm deep. (F) The normal strain distributions, *ε_xx_* and *ε_yy_*, of the membranes obtained from DIC analysis in both chambers when they are subjected to 8% strain in the *x* direction. The black areas indicate the unobtainable areas of the strains through the image processing. (G) The normal strain *ε_xx_* in the *x*-direction along the *y*-axis of the membrane when the chamber is subjected to 8% strain. The coordinate system is shown in F. The red and blue solid lines represent the normal strain *ε_xx_* of the newly developed chamber and the commercial chamber, respectively. (H) Comparison of the standard deviations in terms of strains, *ε_xx_* (left figure) and *ε_yy_* (right figure), between the newly developed chamber and commercial chamber, when the chambers are subjected to 8% strain. The standard deviations must be decreased if the chambers are subjected to less than 8% strain.

Fig. 1F shows representative normal strain distributions, *ε_xx_* and *ε_yy_*, of the membrane in the PDMS chamber developed in this study and a commercially available PDMS chamber calculated by DIC when the chamber was subjected to 8% strain in the *x* direction. The representative normal strain distribution *ε_xx_* along the center line of the chamber is exhibited in Fig. 1G. The uniformity of the strain distribution in the newly developed chamber (red in Fig. 1G) showed improvements over that of the commercial chamber (blue in Fig. 1G), which was attributed to differences in the applied loading type (concentrated load versus uniformly distributed load, respectively) (Fig. 1F). Fig. 1H presents the quantitative differences in this improvement, where the longitudinal axis represents the standard deviations of the strain on the membrane (left figure: *ε_xx_*, right figure: *ε_yy_*). The improvement of the standard deviation in *ε_yy_* was ascribed to the thicker sidewalls of the new PDMS chamber (5 mm) compared with the commercial chamber (2.5 mm) (Figs. 1A–D). Consequently, the new PDMS chamber achieved an approximately 50% improvement in terms of strain homogeneity compared with the commercial chamber. In the new chamber, the strain distribution of the cell-culturing area was 7.97±0.22% (mean±s.d.) under an applied strain of 8%. The homogeneity of the mechanical force applied to the cells was guaranteed by two mechanisms: the suppression of cell reorientation behavior due to the microgrooved membrane, and the reduction in the strain variability of the membrane due to the use of the new chamber. In other words, inhomogeneities caused by the cellular orientation and the strain distribution of the membrane lead to fluctuations in gene/protein expression in strained cells (Morita et al., 2018), resulting in inaccurate cell differentiation assessments.

**Fig. 2.**
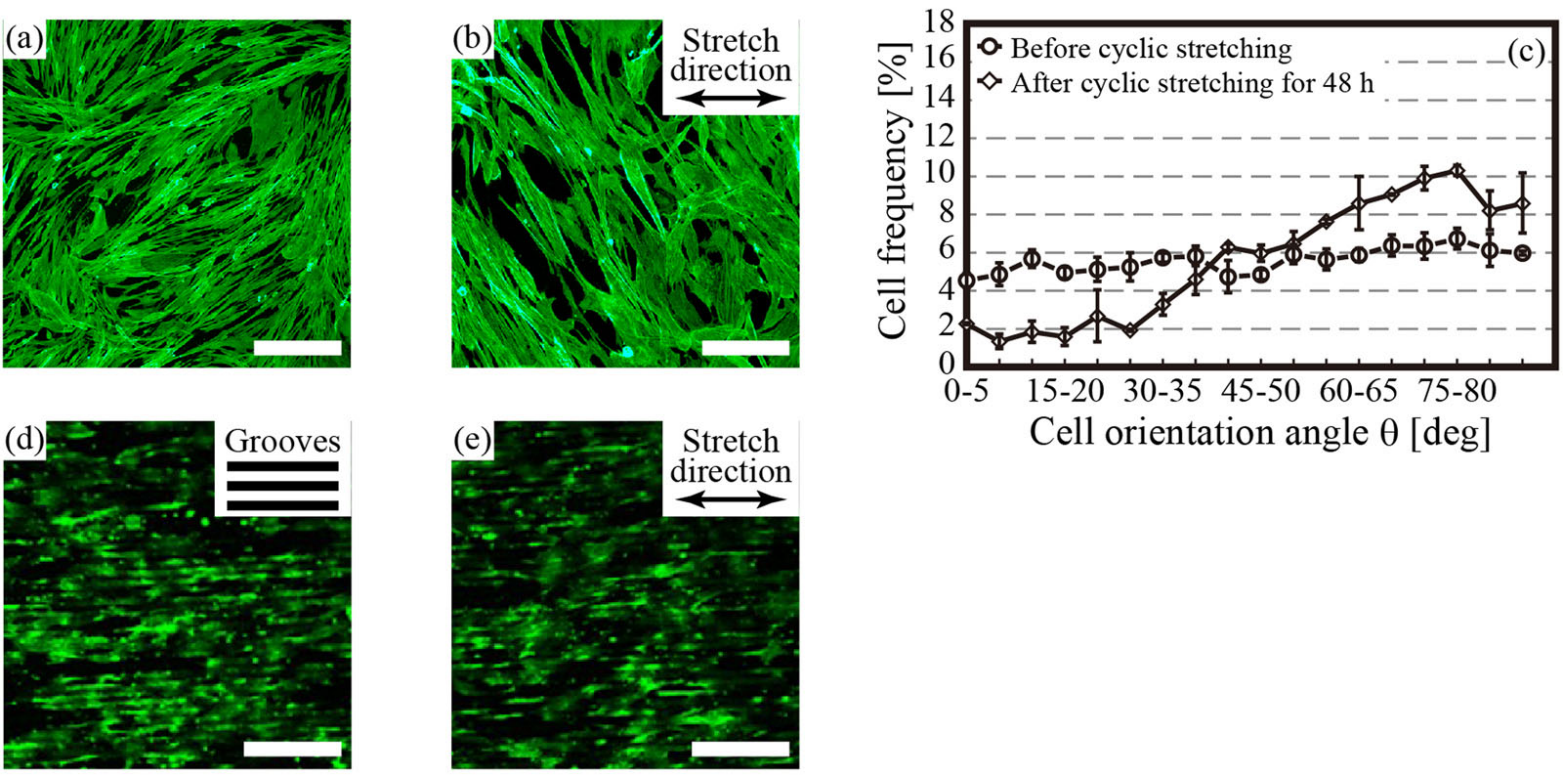
**Fluorescent images indicating the cytoskeleton of human bone marrow-derived mesenchymal stem cells (hBMSCs) subjected to cyclic stretching.** Stretch ration: 8%, cyclic frequency: 1 Hz. (A,B) The hBMSCs were cultured on the plane PDMS chamber before cyclic stretching (A) and after cyclic stretching for 48 h (B). The cells avoided the cyclic stretching by aligning nearly orthogonal to the stretch direction. (C) The quantitative result of the escape of the hBMSCs cultured on the plane PDMS chamber from the cyclic stretching. The cell orientation angle *θ* means an angle between the stretch direction and the longitudinal axis of each cell when the cells were considered as ellipses. (D,E) The hBMSCs were cultured on the aligned microgrooved PDMS chamber before cyclic stretching (D) and after cyclic stretching for 48 h (E). The microgrooves were aligned horizontally, and the cells were oriented along the direction of the microgrooves. Scale bars: (A,B) 100 µm, (D,E) 200 µm.

### Relationship between culture membrane strain and hBMSC mRNA expression

The cells cultured on the plane PDMS chamber were realigned to specific angles to the stretch direction (Fig. 2A–C) although the cells were oriented along the lines of the microgrooves in the PDMS chamber before and after cyclic stretching (Fig. 2D–E), as indicated by the cytoskeleton of the cells based on fluorescein isothiocyanate staining (Enzo Life Sciences, New York, NY, USA).

**Fig. 3.**
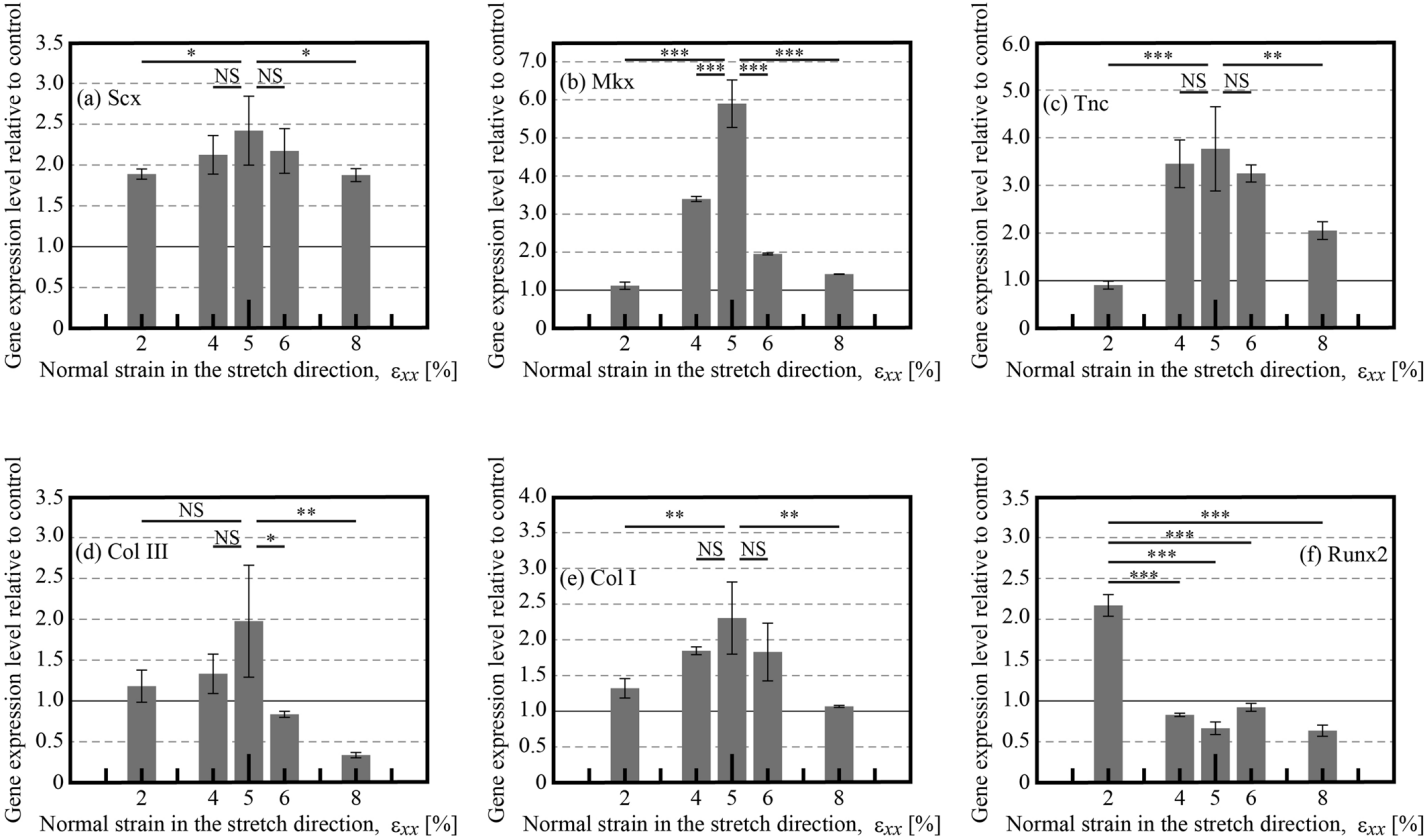
**mRNA expression levels under normal strain in the stretch direction, *ε_xx_*, of the newly developed elastic PDMS chamber.** (A–F) show *Scx*, *Mkx*, *Tnc*, *Col III*, *Col* *I* and *Runx2* expression, respectively. Data are normalized to the corresponding mRNA levels in unstretched cells on the plane elastic PDMS chamber (defined as 1). All data are expressed as the means±s.d. *P<0.05, **P<0.01, ***P<0.001. NS, not significant.

**Fig. 4.**
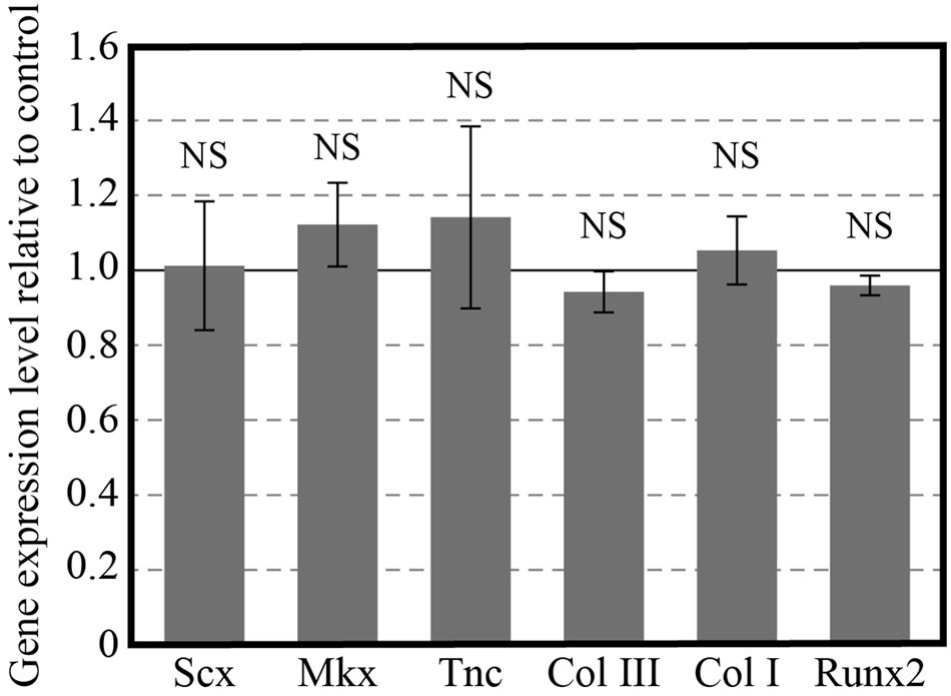
**mRNA expression levels in aligned and unstretched cells on the microgrooved elastic PDMS chamber.** Data are normalized to the corresponding mRNA levels in unstretched cells on the plane elastic PDMS chamber (defined as 1). All data are expressed as the means±s.d. NS, not significant.

Fig. 3 shows the changes in hBMSC mRNA expression under different cyclic strain amplitudes in the newly developed chamber. The expression levels of each mRNA showed different effects. However, the maximum expression levels of *Scx*, *Mkx*, *Tnc*, *Col III*, and *Col I*, which are representative markers of stem cell-to-tenocyte differentiation, were observed at 5% strain. *Mkx* is an important gene at the initial stage of tendon tissue development (Kayama et al., 2016). Meanwhile, *Scx* is a main gene in tendon tissue development and homeostasis (Brent et al., 2003; Kayama et al., 2016; Li et al., 2015). Because both of these key markers showed maximum expressions at 5% strain, it is likely the most effective strain amplitude for differentiation under cyclic stretching. When cells are allowed to undergo reorientation, the most effective under cyclic stretching strain amplitude is almost 8% (Morita et al., 2014, 2015; Nam et al., 2015). The decrease in the most effective strain from 8% in previous studies to 5% in the present study can be ascribed to the suppression of cellular reorientation, which puts cells under a more severe mechanical environment. One might be able to point out that even the fabricated microgrooves of the PDMS chamber must involve an effect on the protein expression levels of the hBMSCs, even if the cyclic stretch stimulus isn't applied. Fig. 4 shows the effect of the microgrooves on the protein expression levels of the hBMSCs. The value of the longitudinal axis is normalized by the protein expression of hBMSCs cultured on the plane PDMS chamber. It can be concluded that the microgrooves of the PDMS chamber do not have any influence on the tenogenic differentiation.

From the perspective of *in vivo* conditions, an effective strain of 5% is reasonable, since it is the same mechanical condition to which *in vivo* tenocytes are subjected. *In vivo* tendon or ligament tissue is usually mechanically elongated with a maximum strain of 4–5% (Bates et al., 2016; Wang, 2006). In addition, tenocytes or ligament fibroblasts inside tissue align their long axis along the stretched direction (Benjamin et al., 2008) and are subjected to mechanical stimulus. Therefore, the maximum mRNA expression levels for the tenogenic differentiation obtained in this study occurred under cellular orientation and strain stimulus conditions that mimicked *in vivo* constructive and mechanical conditions.

When studying the strain conditions for tenogenic differentiation of hBMSCs, cyclic frequency must also be considered. We applied a stimulative cyclic frequency of 1 Hz, because it has been shown to be effective for hBMSC proliferation (Song et al., 2007) and corresponds with the physiological motions of tendon (e.g. walking is nearly 1 Hz) (Smith and Joyce, 2017). The gene expression levels of tenogenic differentiation markers supported the use of 1 Hz as the optimal stimulative cyclic frequency, which the experiment was conducted under 5% strain and 48 h duration (Fig. S1). Conversely, osteogenic differentiation can also be induced when hBMSCs are subjected to cyclic stretching (Chen et al., 2008). Therefore, we assessed *Runx2* expression, one of the primary markers of osteogenic differentiation. The most effective strain condition for osteogenic differentiation was 2% (Fig. 3F), which was in agreement with the qualitative results of Chen et al. (2008), who concluded that a 3% strain promoted hBMSC differentiation compared with a 10% strain under a cyclic frequency of 1 Hz, although cellular reorientation was not constrained.

In summary, the newly developed PDMS chamber, which suppresses cellular reorientation behavior and exerts a homogeneous strain distribution on the membrane, supported the evaluation of tenogenic differentiation of hBMSCs under cyclic stretching. The results showed that the expression of related genes was dependent on the degree of strain, and that a 5% strain was the most effective at promoting differentiation. These results support tendon tissue engineering, which requires effective differentiation induction of stem cells into tenocytes.

## MATERIALS AND METHODS

### Fabrication of a newly developed elastic chamber for cell culturing

A commercially available PDMS chamber for cell culturing with cyclic stretching was obtained. This chamber has thin sidewalls and can apply uniaxial load through support points (Fig. 1A). The thin sidewalls cannot prevent significant contraction in the direction perpendicular to the applied load due to Poisson's effect, and the point load support negatively affects the homogeneity of the normal strain distribution in the applied load direction (Fig. 1B). Therefore, a new PDMS chamber was designed, which has thicker sidewalls and a linear load support (Fig. 1C). The thicker sidewalls diminish Poisson's contraction, and the linear load support provides assurance for the uniformity of the normal strain distribution in the applied load direction (Fig. 1D). First, an aluminum mold was made to fabricate the sides of the PDMS chamber (Fig. S2). Then, a fabricated PDMS membrane with aligned microgrooves (see below) was attached to the bottom surface of the PDMS chamber.

### Fabrication of the stretched membrane with aligned microgrooves

A glass wafer (50.8×50.8×1.0 mm^3^) was covered entirely with photoresist (AZP4903; MicroChemicals GmbH, Ulm, Germany) using a spin-coating method (2000 rpm, 20 s), which was dried in a thermostatic oven at 90°C for 1 h. Parallel lines (10 µm apart, 5 µm deep) were lithographed in a 20×20-mm^2^ central area on the coated glass wafer using a laser lithography system (µPG104-UV; Heidelberg Instruments Mikrotechnik GmbH, Heidelberg, Germany) after drying. The exposed photoresist on the glass wafer was removed by submerging in developer (AZ 400K; AZ Electronic Materials USA Corp., NJ, USA) for 3 min. The wafer was then dried in a thermostatic oven at 90°C for 1 h. Finally, the glass wafer was used as a mold for the fabrication of the aligned microgrooved membrane.

The glass-wafer mold was covered entirely with PDMS solution (base:hardener=10:1) (SYLPOT 184; Dow Corning Toray, Tokyo, Japan) using a spin-coating method (300 rpm, 1 min). The PDMS solution was cured by heating at 100°C for 10 min, and the PDMS membrane (300-µm-thick) was removed from the mold. The PDMS membrane had aligned microgrooves that were 10.01±0.45 µm (mean±s.d.) wide and 5.00±0.12 µm deep (Fig. 1E). Finally, the aligned microgrooved membrane was attached to the bottom surface of the new PDMS chamber.

To compare the deformation distribution of the membrane between the new and commercial PDMS chambers, which were applied uniformly distributed load and concentrated loads, respectively, the strain distribution was measured using a digital image correlation (DIC) method (Sutton, 2008) before cell seeding. Briefly, the DIC method is one of the powerful techniques to determine full-field surface displacements of a measured object from the movement of the inherent speckle patterns on the surface. The in-plane strain distributions in the parallel (*x*) and orthogonal (*y*) directions of the stretch, *ε_xx_* and *ε_yy_*, can be calculated from the displacement information, *u* and *v*, respectively, which are the displacement in each position of the membrane.
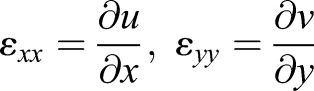
The chambers were cleaned ultrasonically with neutral detergent, tap water, and distilled water, in sequence; placed in sterile phosphate-buffered saline (Cosmo Bio, Tokyo, Japan); and then sterilized by exposure to ultraviolet light in a sterile hood for 1 h. Finally, they were coated with human fibronectin (R&D Systems, Minneapolis, MN, USA) at a concentration of 1 µg/cm^2^ and incubated for 3 h.

### Cell preparation

Human bone marrow-derived mesenchymal stem cells (UE6E7T-3; Riken Cell Bank, Tsukuba, Japan) were cultured in accordance with the protocol of the supplier, and were maintained in low-glucose Dulbecco's modified Eagle's medium (Wako Pure Chemical Industries, Ltd., Osaka, Japan) containing 10% new bone calf serum, 0.5% GlutaMax, and 0.05% gentamicin (Invitrogen, Carlsbad, CA, USA) at 37°C under a 5% carbon dioxide atmosphere in a humidified incubator. All hBMSCs in this study were used at or before the 29th passage to ensure a high proliferative capacity. The hBMSCs were cultured in 25-cm^2^ culture flasks (BD Biosciences, Franklin Lakes, NJ, USA) at an initial density of 1.0×10^4^ cells/cm^2^ for expansion without differentiation. The medium was replaced every 3 days. At near-confluence, occurring every 5–7 days, cells were detached from the culture flasks with 0.25% w/v trypsin (Wako Pure Chemical Industries, Ltd.) with 1 mM ethylenediaminetetraacetic acid (TaKaRa Bio, Shiga, Japan) and seeded into new culture flasks. The trypsinized hBMSCs cultured in flasks were plated onto the bottom of the aligned microgrooved chambers at a density of 1.0×10^4^ cells/cm^2^ and cultured without cyclic stretching for 2 days.

### Cyclic stretching conditions

Table 1 lists the experimental conditions of the study. The hBMSCs cultured on the plane PDMS chambers without cyclic stretching were prepared as the control. And the aligned microgrooved chambers with cultured hBMSCs were set in a mechanical cyclic uniaxial stretching system with applied cyclic stretching in a humidified incubator. Our previous studies, in which cellular reorientation behavior was not constrained during cyclic stretching, suggested that the most effective strain was between 2% and 8% (Morita et al., 2013b, 2014, 2015). Therefore, six strain amplitudes, 0 (control), 2, 4, 5, 6 and 8%, were tested to determine the most effective strain for stem cell-to-tenocyte differentiation. The test was conducted for 48 h, similar to previous studies (Chen et al., 2008; Morita et al., 2013a, 2013b, 2015; Xu et al., 2012), and we confirmed that the gene expression levels of the stretched cells had stabilized by 48 h (Morita et al., 2018).

**Table 1.**
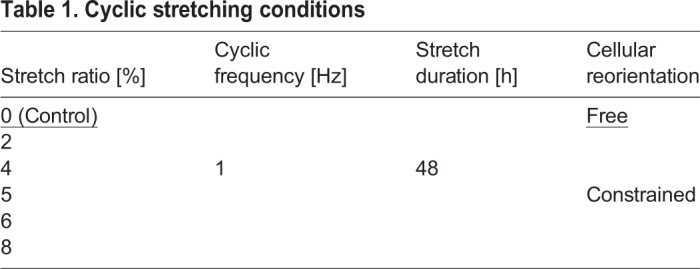
**Cyclic stretching conditions**

### RNA isolation and qRT-PCR

When the cyclic stretching period was completed, the cells were lysed, and their total RNA was isolated using the RNeasy Mini Kit (Qiagen, Düsseldorf, Germany). The purity and concentration of the RNA were assessed based on the absorbance ratio at 260/280 nm. Reverse transcription was performed using a High Capacity RNA-to-cDNA Kit (Applied Biosystems, Carlsbad, CA, USA). Glyceraldehyde 3-phosphate dehydrogenase (*Gapdh*; internal control), scleraxis (*Scx*), mohawk (*Mkx*), tenascin-C (*Tnc*), type III collagen (*Col III*), type I collagen (*Col I*), and runt-related transcription factor 2 (*Runx2*) expression levels were analyzed using pre-designed minor groove binder probes (Applied Biosystems), TaqMan PCR Master Mix (Applied Biosystems), and a Real-Time PCR System (ABI 7300; Applied Biosystems). Gene expression levels were calculated using the standard curve method and normalized relative to *Gapdh* gene expression levels. The primers used for the qRT-PCR are listed in Table S1.

### Statistical analysis

The means±s.d. (s.d.) are reported for five single repeated samples. Unpaired Student's *t*-test for two-group comparisons, and one-way analysis of variance (ANOVA) with Tukey-Kramer test for multi-group comparisons were used for the statistical evaluation, and *P*-values <0.05 were considered to indicate statistical significance.

## Supplementary Material

Supplementary information
