# Supplementary material for: The optimal mechanical condition in stem cell-to-tenocyte differentiation determined with the homogeneous strain distributions and the cellular orientation control
Source: Biol Open. 2019 May 22;8(5):bio039164. doi: 10.1242/bio.039164 (PMC6550065; doi:10.1242/bio.039164)
Supplement: Supplementary information [file biolopen-8-039164-s1.pdf]

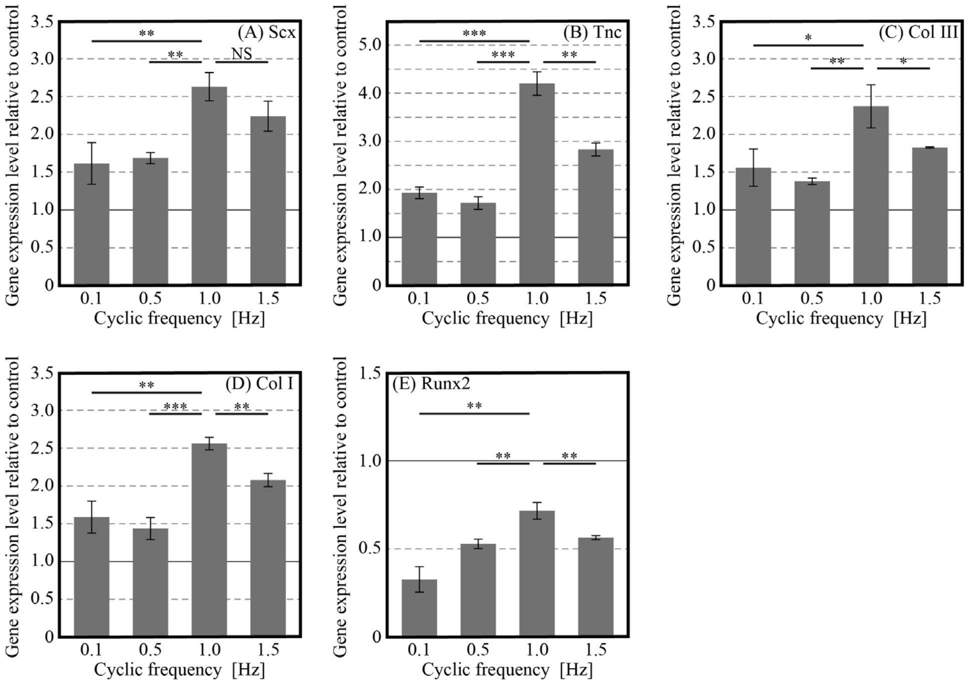

Fig. S1. mRNA expression levels with cyclic frequency of stretching. Stretch ratio: 5%, stretch duration: 48h. (**A**)–(**E**) represent *Scx*, *Tnc*, *Col III*, *Col I*, and *Runx2*, respectively. Data are normalized to the corresponding mRNA expression levels in unstretched cells in the microgrooved membrane (defined as 1). All data are expressed as the means ± standard deviation. *$p < 0.05$; **$p < 0.01$; ***$p < 0.001$. NS, not significant.

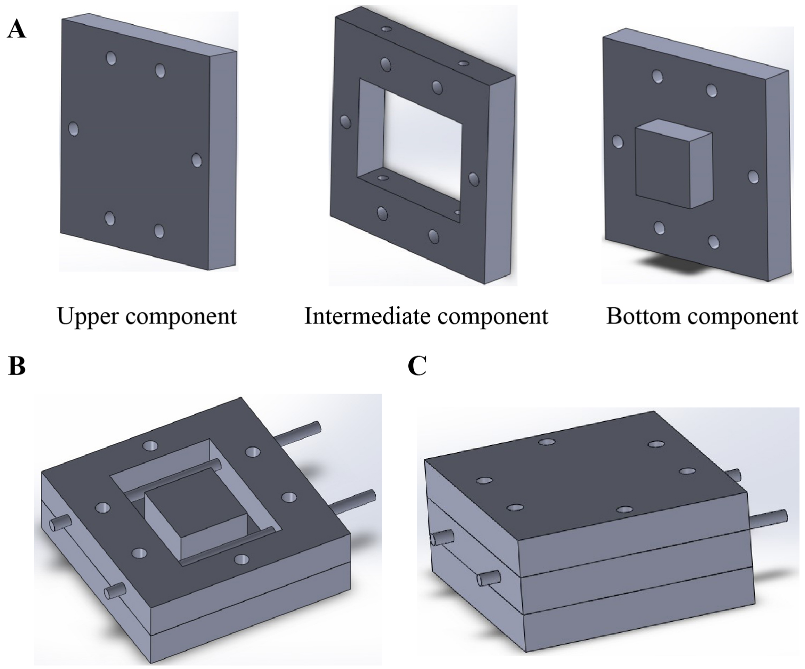

Fig. S2. Aluminum mold used as the side of the new poly(dimethylsiloxane) (PDMS) chamber. (**A**) The three (bottom, intermediate, and upper) components of the mold. (**B**) The bottom and intermediate components are attached, and the rods are used to make holes in the chamber. Then, the chamber is filled with PDMS solution (base:hardener = 10:1) (SYLPOT 184; Dow Corning Toray, Tokyo, Japan). (**C**) The upper component is assembled, and these components are removed after the PDMS has cured.

Table S1. List of the primers used for quantitative real-time reverse-transcription PCR.

Gene	Assay ID	Reference sequence	Assay location
Gapdh	Hs02758991_g1	NM_001256799.2	752
Scx	Hs03054634_g1	NM_001080514.2	620
Mkx	Hs00543190_m1	NM_001242702.1	565
Tnc	Hs01115665_m1	NM_002160.3	2278
Col I	Hs00164099_m1	NM_000089.3	753
Col III	Hs00943809_m1	NM_000090.3	3940
Runx2	Hs00231692_m1	NM_001015051.3	900
